# Cellular Processes and Forces Shaping the Embryo: Lessons from *C. elegans*

**DOI:** 10.3390/cells15070645

**Published:** 2026-04-02

**Authors:** Michel Labouesse, Teresa Ferraro, Flora Llense, Jonathon Heier, Zoe Tesone, Jeff Hardin

**Affiliations:** 1 Development, Adaptation and Ageing (Dev2A)—Institut de Biologie Paris Seine, Sorbonne Université, 7-9 Quai Saint-Bernard, 75005 Paris, France; teresa.ferraro@sorbonne-universite.fr (T.F.); flora.llense@sorbonne-universite.fr (F.L.); 2 Department of Integrative Biology, University of Wisconsin-Madison, 1117 W. Johnson St. Madison, Madison, WI 53706, USA; heier2@wisc.edu; 3 Cellular and Molecular Biology Program, University of Wisconsin-Madison, 1117 W. Johnson St. Madison, Madison, WI 53706, USA; ztesone@wisc.edu

**Keywords:** *C. elegans*, axis extension, dorsal intercalation, ventral enclosure, adherens junction, hemidesmosome, cytoskeleton, rosette, mechanotransduction, α-catenin

## Abstract

Embryo and organ shapes emerge from the interplay between genetic programs and physical forces. In recent years, there has been a growing appreciation of the role of mechanical forces in morphogenesis. Here, we review how the integration of advanced genetic approaches with high-resolution imaging, biophysics, and modeling has begun to yield new insights into *C. elegans* embryonic morphogenesis. Building on past reviews in the field, we analyze dorsal intercalation, ventral enclosure, and axis extension, with a focus on how forces impinge on cellular processes and serve to coordinate morphogenesis across adjacent tissues through mechanotransduction. We also discuss how different forms of cellular rosettes contribute to ventral patterning and head morphogenesis, which had not been discussed in previous reviews. Throughout, we highlight how the reciprocal feedback mechanisms between molecular processes and mechanical forces, as well as cell material properties, shape the embryo.

## 1. Introduction

Embryonic morphogenesis is the set of biological processes by which tissues form, become positioned relative to one another, and by which the embryo acquires its final body shape. This process entails a complex interplay between mechanical and biochemical factors. Understanding how these factors interact during embryonic development is crucial for uncovering the fundamental principles governing embryogenesis, as well as for defining how their dysregulation may lead to disease.

*C. elegans* provides a simple and integrated model for studying the cellular processes and mechanical forces that drive embryonic morphogenesis. Following fertilization and the first cell divisions, the embryo begins gastrulation at the 28-cell stage, roughly 100 min post-fertilization. Cell differentiation starts when epidermal and intestinal cells are born about four hours post-fertilization, at approximately the 320-cell stage [1].

The end of gastrulation is marked by closure of the ventral cleft, after which major morphogenetic movements ensue [2]. These include the intercalation of epidermal cells originating from the left and right sides of the dorsal midline, as well as the ventrally directed epiboly (spreading of a tissue sheet) of the most ventral epidermal cells. The anterior region of the embryo also undergoes substantial morphogenetic rearrangements, including the anterior migration of epidermal cells, the ingression of arcade cells that will form the buccal opening, and involution of anterior neuroblasts. At this stage, the embryo largely ceases cell division and begins to elongate.

Here, we focus on signaling pathways that control actomyosin activity during dorsal intercalation and ventral enclosure. We also highlight the key role played by the formation and resolution of cellular rosettes during late gastrulation and anterior morphogenesis. Although the final step of ventral enclosure—pocket cell sealing—also involves rosette formation and resolution, we discuss this process in the section devoted to ventral enclosure. Finally, we discuss mechanotransduction processes at adherens junctions and between muscle and epidermis during elongation, which involves the dramatic coordinated change in dimensions of epidermal cells to transform the embryo from a bean to a vermiform shape. Whenever possible, we highlight the interplay between physical parameters and cell biochemical processes.

Although cell–cell adhesion complexes play important roles at many stages of early development, we postpone discussing them in detail until after introducing the major morphogenetic events of *C. elegans* embryogenesis at the cellular level. One of these events is embryonic elongation, during which cell–cell adhesion plays key mechanical roles and cellular mechanics becomes prominent, influencing junction composition. Note that we mention the names of the closest mammalian homologs of *C. elegans* proteins discussed in the text when first mentioned in a given section; Table 1 also gives the mammalian name equivalent of proteins displayed in the figures. Table 2 summarizes the temporal order of the events discussed in the review. Box 1 lists the main abbreviations and provides a short lexicon of physical terms. Box 2 summarizes the main novel approaches that have been developed to study embryos in the past ten years.

We do not address early gastrulation, epithelial polarity, the role of the apical extracellular matrix, or microtubule organization, all of which can influence epidermal morphogenesis and have been reviewed elsewhere [3,4,5,6]. Similarly, we do not cover the morphogenesis of other tissues and refer the reader to recent reviews on these topics [7,8,9,10,11].

## 2. Unexpected Twists on Actin Regulation During Dorsal Intercalation and Ventral Enclosure

### 2.1. Dorsal Intercalation

Dorsal intercalation is the earliest epidermal morphogenetic process, occurring soon after epidermal cell birth and before adherens junctions are fully mature. How this process is initiated remains unclear, in part because transcription factors associated with dorsal intercalation phenotypes also act at earlier stages, making it difficult to distinguish direct from indirect effects [12,13]. During dorsal intercalation, epidermal cells from the left and right sides of the embryo extend medial protrusions and migrate to interdigitate, after which they begin to fuse (for recent reviews, see [10,14]). Early drug studies showed that both actin and microtubules are required for dorsal intercalation [15].

**Figure 1:**
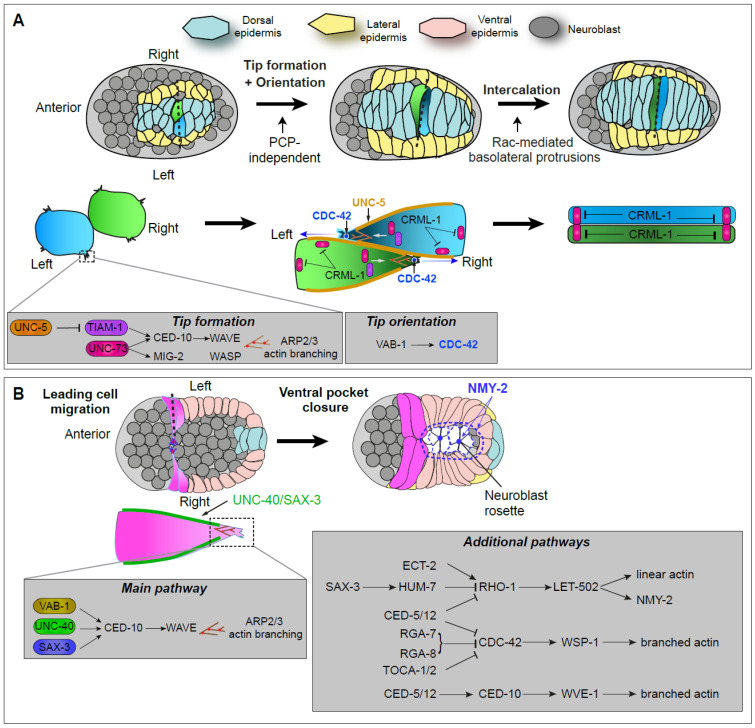
Pathways regulating dorsal intercalation and ventral enclosure. (**A**) Top: dorsal view of the embryo at three stages of dorsal intercalation. Middle: pairs of color cells from top (rotated 90°), showing the distribution of key regulators. The transmembrane proteins VAB-1 and UNC-5 are expressed in the dorsal epidermis. Bottom: main pathways involved in tip formation and tip orientation. (**B**) Top: ventral views of an embryo at two steps of ventral enclosure (fuchsia, leading epidermal cells; pink, ventral epidermis pocket cells; blue, dorsal epidermis; gray, neuroblasts). Middle: transverse view along the dotted line of a leading cell (rotated 90°), with the distribution of some markers indicated. Bottom: main pathways involved in tip formation and tip orientation. The transmembrane receptors VAB-1, UNC-40, and SAX-3 are active in ventral neuroblasts; VAB-1 and UNC-40 are also present in the ventral epidermis. In this and the following figures, dorsal epidermal cells are blue, seam epidermal cells are yellow, and ventral epidermal cells are pink.

Using an actin reporter and genetic manipulation of the Rac GTPases CED-10/Rac and MIG-2/RhoG, several regulators of dorsal intercalation have been identified [16,17,18]. Genetic epistasis analyses showed that CED-10 and MIG-2 act redundantly to promote branched actin polymerization via the nucleation-promoting factors WVE-1/Wave and WSP-1/Wsp, respectively (Figure 1A). A third small GTPase, CDC-42/Cdc42, is also required to orient dorsal cell protrusions and ensure proper intercalation; loss of CDC-42 or components of the PAR-6/PAR-3 complex (Pard6/Par3 in mammals) causes two cells from one side to intercalate as pairs (ipsilateral comigration) [16]. Two guanine-nucleotide exchange factors (GEFs), UNC-73/Trio and TIAM-1/Tiam1, function upstream of these GTPases during dorsal intercalation, with TIAM-1 acting specifically on CED-10 [17,18] (Figure 1A).

Given that UNC-73 is uniformly distributed within dorsal epidermal cells, it alone cannot account for the polarized F-actin protrusions extended by dorsal cells. The capping-protein inhibitor CRML-1/Carmil is polarized to lateral regions of dorsal epidermal cells, and later transiently localizes to cell tips after intercalation, where it may influence polarized actin dynamics. CRML-1 may inhibit further tip formation once intercalation completes (Figure 1A, right). Further upstream, axon-guidance receptors regulate protrusive activity: the netrin receptor UNC-5/Unc5 localizes to medial edges of dorsal cells, where it may inhibit TIAM-1, whereas the Eph receptor homolog VAB-1 is necessary for medial enrichment of CDC-42 activity [16,18]. Consistent with this model, *unc-5* and *vab-1* mutants phenocopy the dorsal misdirection observed in *cdc-42* loss-of-function mutants. The ligands acting upstream of VAB-1 and the CDC-42 GEF acting downstream remain unknown, as the loss of obvious candidates fails to result in dorsal intercalation phenotypes or leads to pervasive early defects.

Like other epidermal morphogenetic processes in *C. elegans*, dorsal intercalation requires axon-guidance receptors (see Section 2.2 below) and exhibits extensive functional redundancy. What is less clear is what effectors lead to mediolaterally polarized movement. Dorsal intercalation appears to be similar to other mediolaterally directed cell rearrangements, collectively known as convergent extension, which corresponds to the narrowing of a tissue in one dimension with concomitant lengthening in the orthogonal dimension via directed cell rearrangement. Although the dorsal epidermis is an epithelial tissue, dorsal intercalation may bear closer resemblance to mesenchymal migration driven by basal protrusions (reviewed in [19,20,21]), perhaps because it is not yet a fully differentiated epithelium with fully formed junctions. Dorsal intercalation, thus, contrasts with epithelial intercalation processes that rely on myosin-II and junction remodeling (reviewed in [22,23]). Despite similarities to deep-cell convergent extension, to what extent dorsal intercalation relies on a coordinated orientation of cells within the plane of a tissue sheet—also known as planar cell polarity (PCP) signaling—is not clear. The transmembrane protein VANG-1/Van Gogh has been reported to be asymmetrically localized to the posterior boundaries of some dorsal epidermal cells, a distribution lost in *dsh-2*/Dishevelled mutants [24]. One unpublished report also suggests occasional ipsilateral comigration of dorsal epidermal cells in a minority of *vang-1* mutants [25]. Mild phenotypes in mutants for two Dishevelled homologs (*mig-5*, in addition to *dsh-2*), however, indicate that at least some canonical planar cell polarity pathway components are largely dispensable [26,27], suggesting that dorsal intercalation may differ from another convergent extension-based movement in *C. elegans*, ventral nerve cord formation [26] (see below).

### 2.2. Ventral Enclosure

Ventral enclosure starts when two anterior ventral epidermal cells on each side of the embryo, known as leading cells, extend ventrally and meet to establish new junctions at the ventral midline, pulling the rest of the epidermis ventrally as they do so. Ventral enclosure concludes when more posterior cells, known as pocket cells, cover the remaining unenclosed ventral region. This process combines epiboly with a purse-string-like mechanism [28]. Early cellular and genetic studies identified a central role for branched actin in driving ventral enclosure [28,29,30,31], as well as the axon guidance receptors SAX-3/Robo, UNC-40/DCC, and VAB-1/Eph, which activate CED-10/Rac upstream of the WAVE complex [32]. In addition, an Ena/VASP-mediated filamentous actin network mediates the consolidation of junctions at the midline; junctional components appear to be preloaded in migrating cells, allowing them to be rapidly deployed in a process termed “filopodial priming”, where thin needle-like actin-based protrusions lead the migration [33,34,35]. Adhesion of epidermal cells at the ventral midline depends on the cadherin–catenin complex (CCC), which contains a single classical cadherin, HMR-1A/E-cadherin, an adhesion-specialized β-catenin, HMP-2, and HMP-1/α-catenin (see the full discussion of the CCC in Section 4.2, Section 4.3, Section 4.4 and Section 4.5; [36], reviewed in [37]).

**Figure 2:**
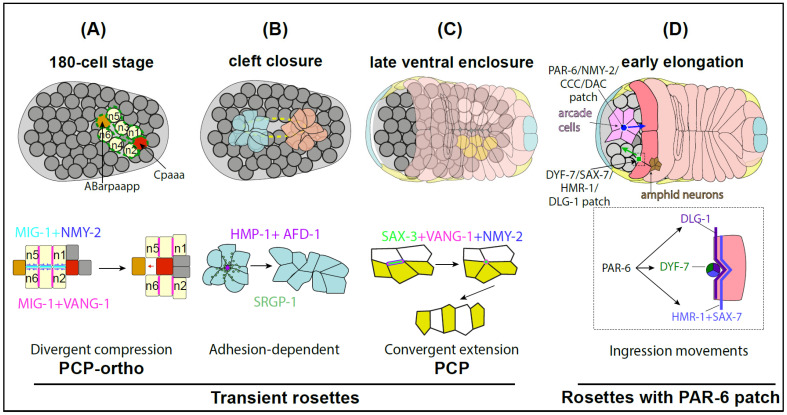
Rosettes during morphogenesis. (**A**–**D**) Four types of rosettes. Top: ventral views of embryos at different stages. Bottom: main proteins involved in rosette formation and/or resolution. Colored cells in the top row indicate those involved in rosette formation; gray cells, precursors. In embryo (**C**), the ventral epidermis (pink cells) has been left partially transparent to reveal two sets of underlying neuroblasts involved in rosette formation (yellow and white). In embryo (**D**), roughly the same stage), the epidermis is left opaque, hiding internal cells. (**A**) Cell Cpaaa on the ventral surface forms successive rosettes with its neighbors (n1–n6), first with cells n1-n4, then with cells n3-n6, ultimately becoming adjacent to ABarpaapp. (**B**) Two rosettes seal the ventral cleft (yellow dotted line) on the ventral surface. (**C**) Five neuroblasts from each side of the embryo (white and yellow cells) move to the ventral midline, where they form two consecutive rosettes; their resolution through convergent extension generates the future ventral nerve cord. (**D**) On the anterior part of the embryo, arcade cells form a rosette with cell bodies ingressing up to 5 µm (blue arrow), whereas amphid neurons on each side form two other rosettes (only one amphid represented), with their tips attached to a patch (green) at the anterior margin of the most anterior epidermal cell (dark pink), which are migrating anteriorly to cover the head (green arrow).

Two recent studies addressed the contribution of neuroblasts to the final purse-string process, which is driven by an actomyosin ring within a group of ventral cells leading to contraction, while others examined signals activating WAVE-dependent branched actin assembly [38,39,40,41,42,43]. High-resolution imaging indicates that non-muscle myosin forms foci at the margins of epidermal pocket cells and in underlying neuroblasts [38,43] (see Box 2). Genetic analysis further demonstrated that NMY-2 (Myh9/Myh10 in mammals) accumulation requires the RHO-1/LET-502/myosin-II pathway in both cell types and synergizes with adherens junction components. Although it was known that neuroblasts influence ventral enclosure [44,45,46], this work revealed that neuroblasts arrange themselves in two transient rosettes that undergo myosin-induced shape changes (Figure 1B; rosettes are discussed in more detail below and described in Figure 2). While these findings do not exclude a signaling role for neuroblasts, they suggest that neuroblast shape changes mechanically promote purse-string formation. This is supported by loss of function for *ani-1*/anillin, which is only expressed in neuroblasts. ANI-1 loss leads to defects in neuroblast division, as well as to defects in purse-string closure of the ventral epidermis [38]. Such a role would be analogous to the role of the amnioserosa during *Drosophila* dorsal closure or of myofibroblast contraction during wound healing [47,48].

Additional regulators downstream of axon guidance receptors and upstream of the small GTPases involved in ventral enclosure include RGA-8/SH3BP1, CED-5/CED-12, Dock/Elmo, and HUM-7/Myo9 [40,41,42] (Figure 1B). In contrast to *ced-10* or *wve-1* mutants, which fail to migrate altogether due to severely reduced actin levels at the migration front, loss of these regulators increases F-actin levels and, in some cases, protrusion rates, but causes only weakly penetrant defects, indicating substantial redundancy. Biochemical, genetic, and molecular analyses suggest that HUM-7 acts as a RhoGAP for RHO-1, RGA-8 for CDC-42, whereas CED-5/CED-12 regulates both RHO-1 and CDC-42 [40,41,42]. The F-BAR-domain RhoGAP SPV-1/RGA-7/ArhGAP29 acts as a spatial regulator of CDC-42 and F-actin, promoting their activity away from the leading edge, while inhibiting them at the leading edge and newly formed junctions [39]. Both RGA-7 and RGA-8 appear to antagonize WSP-1, a CDC-42-regulated branched actin nucleation factor, and its partner, the F-BAR proteins TOCA-1/2 (Fnbp1 in mammals), to function in parallel to the LET-502/Rock pathway [49].

Future studies will be needed to define whether these regulators act primarily in leading cells during migration, during the establishment of new junctions between contralateral leading cells, and/or during the purse-string phase. For instance, work on RGA-7 indicates that it may affect junction establishment, potentially through effects on actin and junctional organization similar to the RhoGAP PAC-1/ArhGAP21, which is required to maintain junctional F-actin and adherens junction organization [50] (see Section 4.5). Alternatively, the excessive actin polymerization observed in the absence of these regulators may deplete the pool of G-actin available for polymerization at leading-edge tips. Finally, these regulators may control myosin-II activity in pocket cells or the underlying neuroblasts in parallel to the RHO-1/LET-502 pathway.

## 3. Twisting into Position: Sequential Rosettes During Morphogenesis

Our understanding of late gastrulation events and anterior morphogenesis has lagged behind that of early gastrulation and later embryonic stages (for reviews, see [3,10,14]). Recent studies are bringing new insights to bear on these processes, all of which involve the formation of cellular rosettes. Multicellular rosettes are structures in which at least four cells converge at a single vertex. Planar rosettes can arise through collective apical cell constriction, as during zebrafish lateral line migration, or through planar-polarized constriction, as in the *Drosophila* germband, and typically require myosin-II for their formation and resolution [51,52,53]. In some systems, such rosettes require the planar cell polarity (PCP) pathway, and their formation is a prelude to convergent extension [53]. Other rosettes are truly three-dimensional and involve more complex changes in position as rosettes resolve [54]. *C. elegans* embryos reflect this range of possibilities.

### 3.1. Sequential Rosettes

During early to mid-gastrulation (Figure 2A), two cell movements happen simultaneously: cell internalization and left–right patterning through migration. Advanced reinforcement deep-learning lineage analysis [55] revealed that 37 cells undergo long-range migration; migrating cells form either single or up to three consecutive multicellular rosettes [56]. In one example (Figure 2A), the cell Cpaaa forms two sequential rosettes, first with cells n1–n4, then n3–n6, ultimately becoming repositioned next to ABarpaapp.

A genetic screen revealed partially penetrant formation and/or resolution defects in mutants for the two core PCP pathway components, VANG-1/Van Gogh and MIG-1/Frizzled (for a review, see [57]), as well as in the atypical adhesion GPCR homolog LAT-1 (Agdr1 in mammals), known for its role in regulating spindle orientation in early embryos [58]. VANG-1 localizes to vertical rosette edges, whereas MIG-1 is found both at the midline and along vertical rosette edges; together they promote enrichment of the myosin-II heavy-chain NMY-2 at the midline [56]. Compared with canonical PCP-driven convergent extension, this pathway is distinguished by NMY-2 (Myh9/Myh10 in mammals) enrichment specifically at the midline, likely reflecting the complementary localization of MIG-1 and VANG-1 to edges perpendicular to contracting edges, a process Xu and colleagues term “PCP ortho” [56]. Further dissection of this mechanism, particularly the role of LAT-1, will likely require optogenetic tools and fluorescent LAT-1 reporters.

### 3.2. Ventral Cleft Closure

After endodermal and mesodermal cells complete their ingression during gastrulation, a ventral cleft persists and is subsequently closed by the movements of neighboring neuroblasts (Figure 2B). Failure to complete ventral cleft closure, as observed in mutants affecting signaling proteins such as VAB-1/EphR and PTP-3/LAR, compromises further morphogenesis (reviewed in [2,14]). Transient protrusions are observed during normal cleft closure [59], so these signaling proteins may regulate the machinery that steers cell migration; consistent with this possibility, *wve-1*/Wave mutants also display cleft-closure defects [31].

Once neuroblasts have completed their migration, cell adhesion molecules are required to seal the cleft, including components of the cadherin–catenin complex (see Section 4.2, Section 4.3, Section 4.4 and Section 4.5 below). Classic evidence for CCC-dependent adhesion comes from embryos lacking both maternal and zygotic HMR-1/E-cadherin [36] and from double mutants for HMP-1/α-catenin and SRGP-1/srGAP [59], which frequently fail to close the cleft. Consistent with a requirement for cadherin-based adhesion, HMR-1/E-cadherin and its partners are present during early gastrulation and act synergistically with the adhesion molecule SAX-7/L1cam to promote gastrulation movements [3,60].

Recent work extended these observations: neuroblasts from the left and right sides of the cleft assemble into two transient rosettes enriched in HMP-1 and SRGP-1, along with accumulation of the non-muscle myosin NMY-2 (Myh9/Myh10 in mammals) at central rosette foci, which presumably aids rosette formation and/or resolution (Figure 2B) [61]. HMP-1 accumulation at rosette centers normally requires the F-BAR and C-terminal domains of SRGP-1 (see further below Section 4.3 regarding this interaction). A mutant with a constitutively open HMP-1 M1 domain suppresses the cleft-closure defects of *srgp-1* mutants [61]. Since this mutant is unable to bind SRGP-1 [62], this suggests that activating HMP-1 independently of SRGP-1 binding is sufficient for successful cleft closure.

Additional evidence that the cadherin–catenin complex is involved in cleft sealing comes from the HMP-1 genetic interactor AFD-1/Afadin. AFD-1 accumulates at rosette apices, and its loss genetically enhances weak *hmp-1* alleles. Furthermore, mutations weakening the interaction of HMP-1 with F-actin exacerbate *srgp-1* or *afd-1* loss, implying that coupling of the CCC to the actin cytoskeleton is required for stable sealing [61]. While CCC rosettes may represent a general mechanism for tissue sealing following cell ingression, future studies will be needed to determine whether these CCC-dependent rosettes function primarily by promoting new neuroblast–neuroblast contacts or interactions between the surface and internalizing cells, or both.

Subsequent terminal divisions and movements of some of the neuroblasts that engage in rosette formation likely contribute to ventral morphogenesis after cleft closure; these movements are complex and three-dimensional, are not well-understood, and require further study.

### 3.3. Nerve Cord Assembly Through Convergent Extension

Many of the neuroblasts involved in ventral cleft closure are precursors of the ventral nerve cord (VNC) motor neurons (DAs, DBs, and DDs). Initially positioned on either side of the embryo, they must intercalate along the anterior–posterior axis by the time of hatching [1]. Intercalation of VNC precursors occurs through canonical PCP-driven convergent extension (Figure 2C). Based on tissue-specific GFP markers, VNC precursors appear to converge at the midline, forming first an anterior rosette, and then a posterior rosette, composed of cells from both sides of the embryo. These rosettes probably correspond to those described during ventral pocket closure (see Figure 1B). Their resolution drives cell intercalation, resulting in a single anterior–posterior row of ventral cord motoneurons before the onset of muscle twitching [26].

Consistent with a convergent extension mechanism, mutations in the canonical PCP components VANG-1/Vangl1 and PRKL-1/Prickle—previously implicated in neuronal development (for a review, see [57])—disrupt ventral cord neuron positioning. Loss of SAX-3/Robo further enhances the mispositioning phenotype of *vangl-1* and *prkl-1* mutants, suggesting that it acts in parallel. All three proteins, together with the myosin-II heavy-chain NMY-2, localize to shrinking edges of rosettes and later to rosette foci [26] (Figure 2C). Cellular analyses indicate that these factors promote actomyosin activity along edges destined to shrink. Failure to shrink generally leads to persistent or unstable rosettes.

Overall, VNC rosettes appear most similar to the canonical framework of PCP-driven cell intercalation, with the notable exception of SAX-3, whose involvement is unexpected given its role in axon guidance [63], but the high level of lethality in *sax-3* mutants [64] is consistent with functions in ventral enclosure [32].

### 3.4. Anterior Morphogenesis

Until recently, head morphogenesis had been less-extensively studied than embryonic elongation [2]. Early work had described the cellular events by which arcade cells, which form the buccal cavity, connect to the pharyngeal primordium [65], and identified RhoGAP ZEN-4/MKLP-1 and PAR-6 as essential for arcade cell polarization [65,66]. In parallel, studies showed that amphid sensory organs form through retrograde extension of amphid neuron cell bodies after their attachment via the zona pellucida–domain proteins DYF-7 and DEX-1, which localize near the buccal cavity [67].

A remarkable recent study reveals that anterior morphogenesis relies on coordinated interactions among three tissues: neuroblasts, arcade cells, and anterior epidermal cells [68] (Figure 2D). Using diSPIM and confocal microscopy to track all cell contours, ingression of neuroblasts, the formation of a large arcade-cell rosette, and the migration of anterior epidermal cells were shown to occur concomitantly. The rosette tip is enriched in the adherens junction proteins DLG-1/Dlg1 and HMP-1/α-catenin, NMY-2/Myh9/10, and PAR-6/Pard6, consistent with apical constriction driving rosette formation. Rosette formation, in turn, prefigures lumen formation—in this case, formation of the buccal cavity—a common outcome of rosette resolution in other systems [53]. The presence of PAR-6 is also consistent with its known role in arcade polarization [66]. The same markers are also found in subsets of glial cells and neuroblasts that cluster near the future buccal cavity after migrating with the epidermis. Perturbation of any one of these processes delays the others or disrupts the final pattern, indicating strong interdependence [68]. These authors propose that arcade cells further pattern surrounding neuroblasts, and in turn influence epidermal migration.

Future work will be needed to determine how arcade cells instruct neuroblast behavior, and whether signaling pathways controlling ventral enclosure also regulate anterior morphogenesis. From a mechanical perspective, it will also be important to assess whether ventral enclosure mechanically contributes to anterior morphogenesis. Indeed, theoretical work suggests that it generates long-lasting stresses and strains that influence later morphogenetic events (see below and [69]).

A separate study showed that amphid neurons form a rosette with a vertex patch enriched in PAR-6 and DYF-7 [70]. This patch closely associates, on both sides of the embryo, with the leading edge of the most anterior migrating epidermal cell and follows its anterior-directed movement. This interaction is functionally important, as down-regulation of *elt-1*, which specifies epidermal cell fate, impairs epidermal migration and concomitantly blocks the dendrite movement. Adhesion between amphid tips and the epidermis is mediated redundantly by at least four proteins—DYF-7, SAX-7, DLG-1, and HMR-1—present in both tissues. PAR-6 acts upstream of these adhesion molecules, as its depletion reduces the size of the DYF-7 vertex patch and weakens tissue attachment. Together, these results demonstrate that a multicellular amphid rosette migrates anteriorly in coordination with the epidermis through tight inter-tissue adhesion [70].

The numerous occurrences of rosettes during *C. elegans* development highlights their versatility: transient rosettes can bring distant cells together, close clefts, or drive convergent extension, whereas stable rosettes contribute to final organ positioning. Although not all molecular markers have been examined in each context, a general difference between transient and stable rosettes in *C. elegans* may be that VANG-1 is associated with transient rosettes, whereas PAR-6 characterizes stable ones. Whether there are additional differences awaits further investigation. How rosettes not relying on a *bona fide* PCP pathway form and resolve will require further investigation. In particular, how such rosettes mobilize NMY-2 and CCC components, as well as the role of mechanical asymmetries at the tissue level, is an exciting area for future study.

## 4. Mechanical Twists on Elongation, Part 1: The Epidermis During Initial Elongation

**Figure 3:**
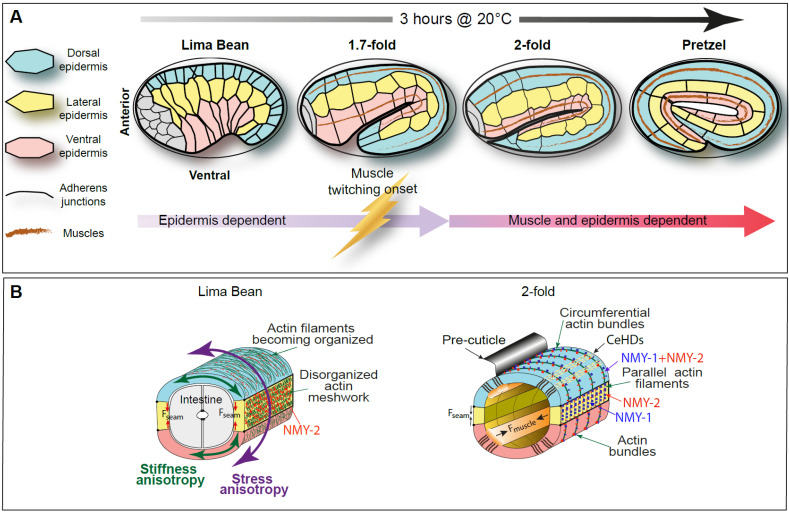
Stages and tissue-level forces during elongation. (**A**) The embryo at four stages of elongation; stages are referred to by their apparent length relative to that of the fertilized egg (50 µm). There are two main phases: (1) lima bean to 2-fold elongation depends mostly on the epidermis, and (2) 2-fold to pretzel also depends on muscles. Between the lima bean and 1.7-fold stages, muscle cells migrate to their final positions and start assembling myofilaments (red line running along the dorsal and ventral epidermis); muscle twitching starts at the 1.7-fold stage. (**B**) Cross-sectional views of the embryo at the lima bean and 2-fold stages, depicting the organization of actin and distribution of non-muscle myosin heavy chains NMY-1 and NMY-2 (for clarity, the intestine has been removed from the 2-fold embryo); the structure of hemidesmosome-like structures CeHDs (light yellow lines) are depicted in greater detail in Figure 5A. Also illustrated are the main mechanical parameters at play at the lima bean stage and the relative orientation of forces originating from muscles and seam cells once muscle become active. NMY-2 is present in all epidermal cells at both stages, whereas NMY-1 expression starts at the lima bean stage and progressively becomes the predominant heavy chain in seam cells.

Once the embryo has completed ventral enclosure and most of its surface is covered by an external epithelium, it begins to elongate. There are two main phases of elongation (Figure 3A). The first phase depends mostly, if not exclusively, on processes occurring within the epidermis. The second phase depends on muscle activity in addition to epidermal processes. Midway through the first phase of elongation, at approximately the comma stage (approximately one hour after the lima bean stage), dorsal epidermal cells fuse together under the control of the EFF-1 fusogen, a worm-specific single-pass transmembrane protein [71,72]. This event may mechanically influence elongation, possibly through the generation of a very large cell, although its precise contribution remains unknown. In this section, we emphasize the mechanical aspects of elongation rather than the roles of individual molecular players, which have been discussed in several recent reviews [10,14,73].

### 4.1. Generating Anisotropic Tension (A Short Lexicon of Physical Terms, Such as Anisotropic, Can Be Found in Box 1)

The first phase of elongation is achieved through a reorganization of the epidermal actomyosin cytoskeleton. There are two main epidermal cell types: dorsal/ventral and seam epidermal cells (blue, pink, and yellow, respectively, in Figure 3), which display distinct cytoskeletal features. Actin filaments are dense and largely unaligned in seam cells, whereas in dorsoventral epidermal cells, they become progressively organized into parallel circumferential filament bundles (CFBs) that insert near apical junctions (CeAJs) as the embryo reaches the 1.7-fold stage [15]. Junctional proximal actin likely lies largely parallel to adherens junctions; by contrast, CFBs insert orthogonally into this junctional proximal network (anchoring of actin bundles at the CeAJs is discussed below and depicted in Section 4.5). The precise organization of actin filaments in each case remains unclear. In particular, the structure of actin in CFBs (e.g., whether component filaments are short or long, and whether they have uniform or mixed polarity) remains unknown. Moreover, the pathways that contribute to the production of this striking, circumferential actin array have not yet been identified. As discussed above, the canonical PCP pathway, which could be predicted to influence this planar orientation, is unlikely to play a major role. An alternative could be that mechanical factors associated with ventral enclosure generate a pre-stress that creates a general anisotropy within the tissue (see Section 7) below). It is also unclear whether and how CFBs are anchored at sites away from CeAJs. It is known that SPC-1/SMA-1 α/β-spectrin heterodimers contribute to organizing actin arrays in the epidermis [74,75], and could serve to anchor CFBs within the apical domain of dorsal and ventral epidermal cells. A candidate for anchorage of CFBs at the level of apical hemidesmosomes (CeHDs; see Figure 3B and discussion below) is the spectraplakin VAB-10; VAB-10B/Macf contains an actin-binding domain [76].

*C. elegans* non-muscle myosin consists of a regulatory light chain, MLC-4 [77,78], an essential light chain, MLC-5 [77,79], and one of two heavy chains, NMY-2 (similar to mammalian Myh9 and My10) or NMY-1 (similar to mammalian Myh10 and Myh11) [79,80]. NMY-2 is present in early embryos, where it drives embryonic polarity and cytokinesis [81,82]. Its levels progressively decline beyond the 1.5-fold stage, although it remains detectable in all epidermal cells [79]. In contrast, NMY-1 accumulates during elongation and becomes strongly expressed in seam cells [79,80] (Figure 3B). The use of temperature-sensitive alleles of *nmy-1* and *nmy-2* revealed that early elongation mainly relies on NMY-2, and that elongation beyond the 2-fold stage requires either NMY-1 or NMY-2 [79]. Although non-muscle myosin is present in the dorsoventral epidermis, it is thought to be largely inactive due to the RhoGAP RGA-2/Arhgap20, which keeps the RHO-1 GTPase—and consequently LET-502/ROCK—inactive in these cells [83]. A recent study using a FRET sensor reporting on the level of MLC-4 phosphorylation suggests that MLC-4 phosphorylation remains high throughout elongation, although this study lacked the resolution to determine in which cells it remains phosphorylated beyond the 2-fold stage [84].

A central feature of *C. elegans* embryonic elongation is the circumferential (i.e., dorsoventral) narrowing of seam cells and their concomitant anterior–posterior lengthening, which is linked to embryo volume conservation. In contrast to other systems undergoing comparable anisotropic shape changes, such as *Drosophila* germband and amnioserosa cells [51,85,86,87], no actomyosin pulses have been reported in seam cells [88]. Several studies have elucidated an alternative mechanism by which actomyosin-mediated forces lead to embryonic elongation.

In *C. elegans*, several factors are likely to generate stress anisotropy along the embryo’s circumference, as revealed by laser ablation of actin filaments [88] (Figure 3B). First, as outlined above, actin filaments in dorsoventral epidermal cells are predominantly oriented along the circumferential axis. This organization of actin generates stiffness anisotropy, as established by actin laser-cutting experiments, which revealed that the epidermis is stiffer along the DV direction and more compliant orthogonally to actin bundles (Figure 3B; see also Box 2). Second, non-muscle myosin is enriched in seam cells (Figure 3B), likely generating an imbalance of tension between seam and non-seam cells. The most likely scenario is that myosin-II in seam cells creates tension by acting on the disorganized seam actin network, thereby producing anisotropic stress that is propagated along the circumference. In this respect, the *C. elegans* embryo resembles the *Drosophila* germband, which also relies on stress anisotropy for elongation, although this anisotropy arises through distinct molecular and physical mechanisms involving apical actomyosin flows and effectors of segment polarity genes [86,89,90].

Continuum mechanical modeling supports the view that stress anisotropy, stiffness anisotropy, and hydrostatic pressure, together with embryo volume conservation due to the lack of food ingestion, can account for the first phase of *C. elegans* embryo elongation [88]. In this sense, actin bundles in dorsoventral epidermal cells may act as a “molecular corset”, mechanically constraining the embryo and making *C. elegans* embryos similar to other systems—such as plant cells or *Drosophila* eggs—in which networks of oriented fibrils guide elongation [91,92].

### 4.2. Withstanding Anisotropic Tension

To withstand the rigors of morphogenesis, cells must reinforce their adhesions to one another in response to increasing force. Embryonic elongation is a case in point: actomyosin-mediated tension generated during the early phase of elongation places dorsal-seam and ventral-seam epidermal junctions under tension (see Figure 3A). Since tension is anisotropically generated during embryonic elongation, the embryo must withstand particularly high tension at specific seam/non-seam junctions in the epidermis during this time.

**Figure 4:**
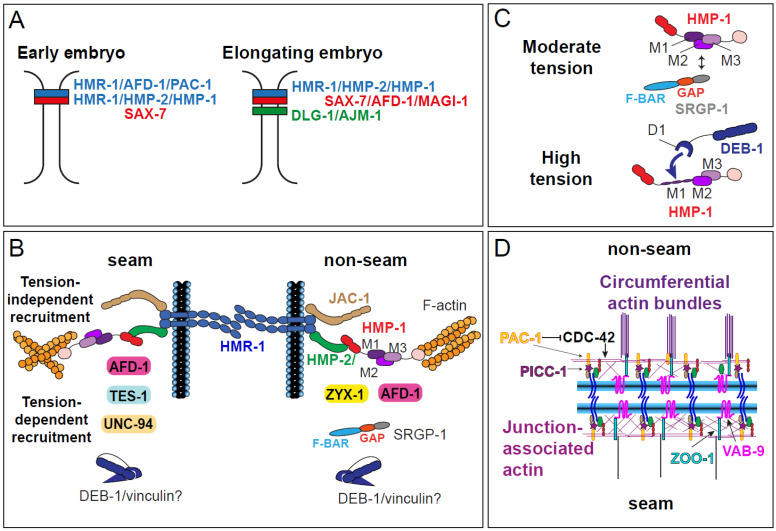
The dynamic anatomy of epidermal junctions. (**A**) The *C. elegans* apical junction (CeAJ) has a different composition in early embryos and elongating embryos: the cadherin–catenin complex (CCC) is present at both stages, with a subset including SAX-7; the DLG-1/AJM-1 complex (DAC) is present only in more mature tissues, where it is basal to the CCC. (**B**) In addition to the core CCC components (HMR-1/E-cadherin, HMP-2/β-catenin, HMP-1/α-catenin, JAC-1/p120catenin), the CCC can include different additional proteins depending on the type of tissue and level of tension (see text and Table 1). (**C**) Single-molecule tweezing experiments (see Box 2) indicate that high tension can induce conformational unfolding of HMP-1, exposing a binding site for the D1 domain of vinculin in vitro; in vivo experiments indicate SRGP-1 cannot bind open forms of HMP-1. (**D**) Presumptive organization of actin filaments at junctions, and proteins involved in their anchoring or regulation.

The *C. elegans* embryonic epidermis contains multiple junctional complexes arranged along the apical–basal axis (Figure 4A). Most apical is a *bona fide* adherens junctional complex (CCC; Figure 4B) containing HMR-1A/E-cadherin. The cytoplasmic tail of HMR-1 binds HMP-2/β-catenin, an adhesion-specialized β-catenin; HMP-2 in turn binds HMP-1/α-catenin, which can bind F-actin [93,94]. As mentioned previously, in the early embryo, the L1cam homolog, SAX-7, is also present in some cells, where it acts along with the CCC during gastrulation [60] and rosette formation [70]. In more mature tissues, the DLG-1/AJM-1 complex (DAC) assembles basal to the CCC (Figure 4A) (reviewed in [95]). A middle layer containing SAX-7, AFD-1/afadin, and the membrane-associated guanylate kinase MAGI-1/Magi2/3 lies between the CCC and the DAC [96].

Two key events critically depend on the CCC. As mentioned previously, zygotic loss of HMR-1 leads to the Hammerhead phenotype due to ventral enclosure failure. Zygotic loss of HMP-1 or HMP-2 leads to elongation failure, accompanied by tearing of junctional proximal actin networks and loss of CFB anchoring (the Humpback phenotype [36]).

### 4.3. HMP-1 and Mechanotransduction

As the linker between the CCC and F-actin networks, HMP-1/α-catenin is essential during ventral enclosure and embryonic elongation (see above). Tissue-specific rescue experiments indicate that HMP-1 is required in either seam or non-seam epidermal cells. A FRET-based tension sensor linked to HMP-1 showed that mechanical tension on HMP-1 increases within seam cells during elongation [97] (see Box 2).

How HMP-1 bears mechanical loads has recently been investigated in detail (Figure 4B,C). First, classical structure–function and biochemical experiments indicated that HMR-1/E-cadherin, HMP-2/β-catenin, and HMP-1 form a tripartite complex with conserved regulatory interactions [93,94,98]. Magnetic tweezing experiments have subsequently quantified the mechanical strength of the HMP-2/HMP-1 interface, demonstrating that its phosphoregulation modulates HMP-2/HMP-1 load-bearing capacity [99].

Second, α-catenins can also respond to mechanical load like entropic springs, reversibly unfolding their middle domain (M) under tension and reversibly refolding when tension is no longer present. It also allows tension-dependent recruitment of other proteins to adherens junctions when tension increases, allowing them to bear increasing mechanical load [100,101,102,103]. Unfurling of the vertebrate α-catenin M domain exposes a binding site with the M1 domain for the D1 domain of vinculin [104,105], which allows reinforcement of the connection of the CCC to F-actin. Single-molecule magnetic tweezing experiments indicate that physiologically relevant tension applied to mammalian αE-catenin can expose the vinculin binding site [106].

The *C. elegans* HMP-1 M domain displays properties similar to its vertebrate counterparts, and recent studies have shown that different tension-dependent conformations of the M domain recruit different binding partners (Figure 4C). While the M domain is not strictly necessary for rescuing *hmp-1* loss-of-function mutants, it is likely important for fine-tuning the mechanoresponse: an HMP-1 mutant protein lacking most of the M domain rescues poorly [107]. The M domain plays a similar fine-tuning role in *Drosophila* α-catenin [108]. The mechanical properties of the HMP-1 M domain have recently been examined using magnetic tweezers (see Box 2). Extension of the M domain requires somewhat greater force than for vertebrate αE-catenin but still lies well within a physiologically relevant force regime. Destabilizing salt bridges within the M domain leads to a more extensible, constitutively open M domain. Like vertebrate αE-catenin, HMP-1 can bind the D1 fragment of vinculin when under tension, and D1 binding stabilizes an extended conformation of HMP-1 [99]. While the HMP-1 M domain can bind DEB-1/vinculin [109], a role for DEB-1 in the epidermis has not been reported; its well-documented role is in integrin-dependent adhesion in muscle [110].

As described earlier, the Slit/Robo GAP SRGP-1 participates in ventral cleft closure [61], but was originally identified as a genetic interactor of *hmp-1*, independent of its GAP activity [59]. Subsequent work showed how SRGP-1 functions at junctions [62] (Figure 4B). SRGP-1 binds the HMP-1 M domain via its C terminus, which is sufficient for targeting to CeAJs. Loss of SRGP-1 increases HMP-1 mobility and reduces its junctional levels. Because SRGP-1 function requires its membrane-binding F-BAR domain, it likely stabilizes CCC association with junctional membranes. Surprisingly, the SRGP-1 C terminus is not recruited to CeAJs in an *hmp-1* mutant with a constitutively open HMP-1 M1 domain or in embryos that cannot elongate. This suggests that, under tension, HMP-1 adopts multiple conformational “gears”: SRGP-1 is recruited when HMP-1 is under tension but not fully extended, likely early in elongation, whereas other interactors are recruited when the M domain is unfurled as elongation proceeds (Figure 4B).

Several studies suggest one other way in which α-catenins are mechanically responsive: they can strengthen their attachment to F-actin when they are pulled on, rather like a Chinese finger trap, a phenomenon known as a catch bond (see Box 1) [111,112,113]. Like other α-catenins, the C terminus of HMP-1 mediates actin binding [114,115], and it is likely that HMP-1 also displays catch-bond behavior, but this has not yet been experimentally confirmed.

### 4.4. Non-Uniformly Localized Junctional Stabilizers

Genetic and RNAi-based screens identified several junctional proteins that act alongside the CCC during elongation (Figure 4B,D). These proteins fall into two broad categories. One group is actin-binding proteins that are recruited to epidermal junctions in a non-uniform manner, mainly localizing to seam-dorsal and seam-ventral boundaries in the epidermis. This localization is notable given the anisotropic stress present during elongation (see above), suggesting these proteins may reinforce seam/non-seam junctions. One example is the minus-end actin capping protein UNC-94/tropomodulin, which shows some enrichment at seam/non-seam epidermal junctions. In vitro assays suggest that UNC-94 promotes the formation of HMP-1-dependent actin bundles, perhaps by inhibiting subunit loss from filament minus ends [116]. Future in vivo studies will be needed to explore how UNC-94 contributes to junctional integrity.

The clearest examples of non-uniformly localized junctional proteins, however, are the LIM domain-containing repeat (LCR) proteins ZYX-1/Zyxin and TES-1/Tes. During elongation, both are recruited to seam/non-seam junctions, but show complementary distribution: TES-1 in seam cells, and ZYX-1 in dorsoventral epidermal cells. *tes-1* and *zyx-1* mutants display junctional F-actin defects that are strongly enhanced in hypomorphic CCC mutant backgrounds [117].

LCR proteins are recruited to strained actin filaments in vitro and to stress fibers in cultured cells [118,119], where they are thought to promote the repair of damaged actin filament bundles. They also accumulate at tricellular junctions, which likely experience greater tension than bicellular junctions [120]. The LCR regions of TES-1 and ZYX-1 behave similarly [117], suggesting that these proteins reinforce junctional actin networks under mechanical stress. Consistent with this, recruitment of TES-1 and ZYX-1 is severely compromised in embryos in which elongation fails despite intact CCC components, indicating mechanosensitive recruitment. The mechanism remains unclear and may involve HMP-1-dependent mechanosensing, actin damage during elongation, signaling at junctions, or a combination of these processes [117].

### 4.5. Uniformly Localized Junctional Stabilizers

The second group of junctional stabilizers is uniformly localized at all junctional boundaries. These include MAGUKs ZOO-1/ZO1 [121] and MAGI-1 [96], the divergent claudin VAB-9/Bcmp1 [122], AFD-1/afadin [61,96,123], as well as the Cdc42 GAP PAC-1 and its binding partner PICC-1 (PAC-1-interacting coiled-coil protein 1/CCDC85A-C) [50]. All colocalize or overlap with the core CCC components along the apicobasal axis. Despite their isotropic localization, loss of function for these proteins primarily disrupts seam/non-seam epidermal junctions. While single mutants or RNAi knockdowns are often mild, they strongly enhance morphogenetic defects in the hypomorphic *hmp-1(fe4)* allele, which reduces HMP-1 binding to F-actin [96,115,124]. In some cases (e.g., the MAGUKs), multivalent scaffolding interactions mediated by multiple functional domains likely stabilize CeAJs. VAB-9 may instead limit excessive tensile forces at seam/non-seam cell boundaries through an unclear mechanism [122]. Intriguingly, strong *hmp-1* mutants exhibit HMR-1-containing long filamentous extensions within dorsal cells. These structures are thought to arise when tension from CFBs pulls on the CCC, a phenotype that is reduced in *hmp-1*; *vab-9* double mutants. It raises the possibility that HMP-1 is not solely responsible for anchoring CFBs, and that VAB-9 may contribute to this process. These findings also raise the possibility that CFB retraction in *hmp-1* mutants results from a force imbalance at the seam/dorsal epidermis interface [97].

Like SRGP-1, AFD-1 functions earlier in development, including gastrulation [123] and neuroblast rosette formation [61] (see above), but it is also involved in elongation. AFD-1 localizes to CeAJs [96,125], which depends on the MAGUK MAGI-1 [96]. AFD-1 loss synergizes with hypomorphic CCC mutants [96,125]. Homozygotes carrying a premature stop codon show occasional ventral enclosure and elongation defects, suggesting that AFD-1 stabilizes junctions under tension, although the mechanism remains unclear [125]. In vertebrates, Afadin binds α-catenin via the C-terminal portion of its M domain and stabilizes α-catenin association with F-actin [126,127,128]. Like *Drosophila* Canoe, however, the AFD-1 C terminus appears largely intrinsically disordered (based on AlphaFold predictions; J. Heier, unpublished). In the case of Canoe, a recent report suggests that this disordered domain can bind F-actin [129], raising the possibility that Canoe and AFD-1 could link α-catenin and F-actin, although direct demonstration of binding between AFD-1, HMP-1, and F-actin requires further investigation.

Another isotropic stabilizing system acts via CDC-42. The juxtamembrane region of the cytoplasmic tail of HMR-1 is bound by the p120ctn homolog, JAC-1 [124]. JAC-1 directly interacts with PICC-1, which in turn recruits the Cdc42 RhoGAP PAC-1/ARHGAP2 to specific cell–cell contacts in early embryos, where it acts along with the PAR complex to polarize blastomeres [130]. During elongation, however, PAC/PICC-1/Cdc42 has a mostly PAR-independent role, although the possibility that CDC-42 also works with PAR-6 has not been excluded [50]. A percentage of embryos lacking both maternal and zygotic CDC-42 have elongation defects but show normal PAR polarization. Conversely, CDC-42 overexpression or loss of PAC-1/PICC-1 also disrupts elongation, suggesting that proper CDC-42 activity is needed for cell–cell adhesion and/or actin dynamics during morphogenesis. Loss of CDC-42 could lead to misregulated trafficking of HMR-1, to an overly dynamic junctional actin network, or both, which may impair junction remodeling during elongation.

## 5. Mechanical Twists on Elongation, Part 2: Mechanotransduction Between the Epidermis and Muscle

**Figure 5:**
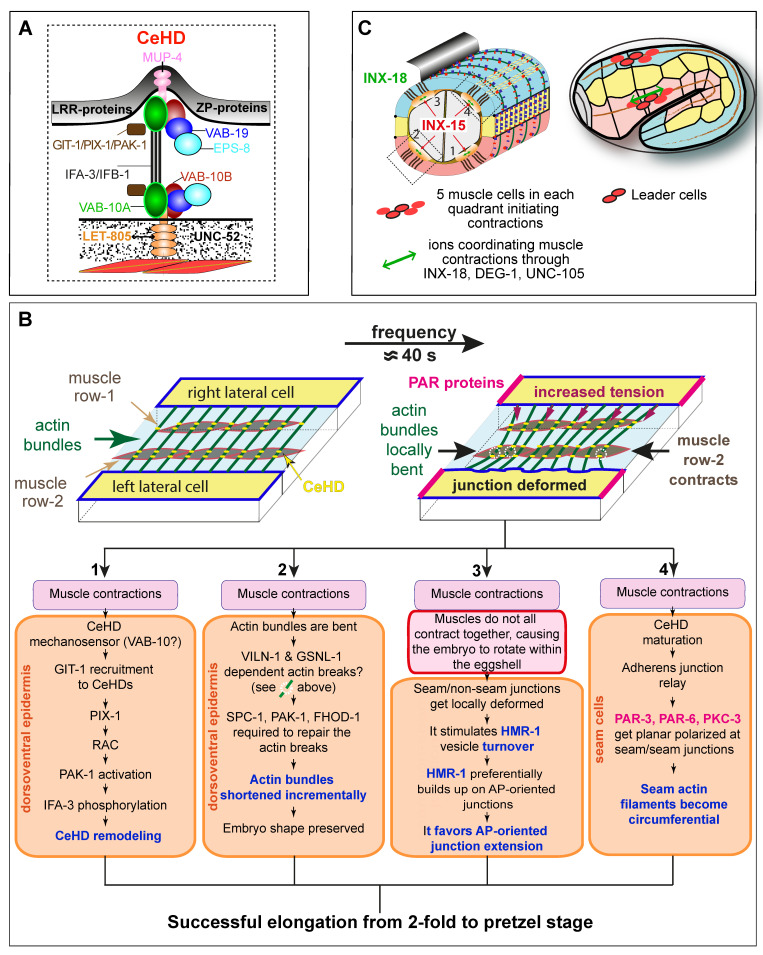
Epidermal cellular processes induced by muscle activity. (**A**) Cross-section through the dorsal epidermis showing a *C. elegans* hemidesmosome-like junction (CeHD; see dotted rectangle and light-yellow lines in panel (**C**)). The CeHD consists of two hemidesmosome-like junctions, one basal and one apical, that are bridged by intermediate filaments (IFA-3/IFB-1, symbolized by the parallel black lines running across the thickness of the epidermis). The apical extracellular matrix is composed of LRR-domain and ZP-domain proteins; the basal extracellular matrix includes UNC-52/Perlecan and additional proteins (for reviews, see [6,10,14,133]). (**B**) The alternating twitching of muscles within a row mechanically modifies the epidermis, which is best evidenced by the local displacement of actin filaments. It likely modifies tension exerted at junctions and deforms junctions during embryo rotations. The consequences on the epidermis are four-fold, promoting: (1) CeHD remodeling and strengthening through mechanotransduction; (2) actin bundle shortening through a partially shared mechanotransduction pathway; (3) HMR-1/E-cadherin turnover and anterior–posteriorly oriented junctional extension through mechanical stimulation; (4) PAR-3/PAR-6/PKC-3 planar distribution and seam actin filament orientation through mechanical stimulation. (**C**) Left drawing: cross-section through a 1.7-fold embryo showing the distribution of the innexins INX-15 in the intestine and INX-18 in muscles. Note also the positions of CeHDs running in the anterior–posterior direction above muscles (multiple yellow dotted lines). Right drawing: position of the muscle cells initiating contractions within each quadrant, two of which play a more prominent coordinating role. The coordination of contraction requires the innexins INX-15 and INX-18, as well as the degenerin/Enac channels UNC-105 and DEG-1.

The second phase of embryonic elongation requires muscle activity, as embryos with inactive muscles fail to elongate beyond the 2-fold stage [131] (Figure 4A). Muscles are tightly connected to the epidermis, and ultimately to the embryonic sheath in embryos (upper gray layer in Figure 5A) or the cuticle in larvae, through two hemidesmosome-like junctions, known as fibrous organelles or CeHDs [132,133] (Figure 5A). Hence, muscle contractions are directly transmitted to the overlying epidermis due to their attachments.

Muscle activity contributes to epidermal morphogenesis through four processes (Figure 5B), which can be visualized by tracking actin lateral displacements and muscle contractions [134,135] (Figure 5B).

### 5.1. CeHD Strengthening

First (Figure 5B, step 1), muscle activity triggers a mechanotransduction pathway within the epidermis, analogous to what has been described at vertebrate focal adhesions [136,137]. Muscle contractions presumably induce a conformational change in a CeHD-associated protein, likely the VAB-10A/plectin [138]. This, in turn, promotes the recruitment of the adaptor and ArfGAP protein GIT-1/Git1 to CeHDs, which, together with the RhoGEF PIX-1/Pix1/Cool2, activates the GTPase CED-10/Rac and, subsequently, the Ser/Thr kinase PAK-1 (homologous to the p21-activated-kinase Pak1). PAK-1 then phosphorylates the core CeHD intermediate filament IFA-3, strengthening CeHDs and enabling their maturation into an adhesion complex capable of withstanding increasing muscle-generated forces [135].

### 5.2. Actin Remodeling

In a second process, muscle activity also contributes to actin remodeling in non-seam epidermal cells (Figure 5B, step 2). As the embryo lengthens during elongation, the circumferential dimension of all epidermal cells decreases due to volume conservation. Consequently, actin bundles within non-seam cells must accommodate this reduction by shortening. Imaging experiments show that muscle contractions can bend actin filaments beyond a threshold known to favor their severing in vitro if actin-severing proteins are present [134,139]. Although individual severing events could not be observed in vivo, two lines of evidence suggest that actin severing occurs in vivo. First, super-resolution microscopy revealed that actin bundles in non-seam epidermal cells are abnormal in *spc-1 pak-1* double mutants, which elongate to the 1.5-fold stage and then retract to the lima bean-like stage once muscle contractions begin. Second, loss of VILN-1, the homolog of the actin-severing protein Villin, suppresses the retraction phenotype of *spc-1 pak-1* double mutants [134]. Based on these observations, it has been proposed that SPC-1, together with PAK-1 and the formin homolog FHOD-1, assists in stabilizing and/or in capping severed actin ends, thereby facilitating the shortening of circumferential actin filaments. Notably, the human FHOD1 protein can promote actin capping, bundling, and nucleation in vitro [140]. This mechanism would promote progressive embryonic elongation and support a viscoplastic deformation analogous to those described in *Drosophila* and mammalian cells (for a review, see [141]).

Although this model is consistent with the genetic data and is supported by a mathematical model of the *C. elegans* embryo as a Kelvin–Voigt material (a material consisting of a spring in parallel to a dashpot submitted to tension), it raises a question regarding whether actin ends can remain in contact following the proposed severing. Indeed, hydrostatic pressure, which builds up during elongation and is thought to be essential for elongation [88,142], could potentially drive severed ends apart, leading to cytoskeletal collapse. One possible way to maintain contact between filament ends might be that FHOD-1, like its vertebrate homolog [140], has capping activity. SPC-1/α-spectrin in a complex with β_H_-spectrin SMA-1 might play a similar stabilizing role, since β_H_-spectrins are actin-binding and crosslinking proteins [143]. It remains unclear whether the activities of these actin-binding proteins are sufficient to prevent filament separation within CFBs.

A final possibility is that non-muscle myosins retain scaffolding functions even when the majority of MLC-4 is inactivated by RGA-2/Arhgap20 in non-seam epidermal cells. Such scaffolding by non-muscle myosins could presumably still operate even if the vast majority of MLC-4 is inactivated in non-seam epidermal cells due to RGA-2 activity. Genetic evidence suggests, however, that *nmy-1* and *-2* are at best minor contributors in this regard. Simultaneous loss of the non-muscle heavy chains NMY-1/Myh10/11 and NMY-2/Myh9/10 after the onset of muscle activity does not induce a retraction phenotype as in *spc-1 pak-1* double mutants [79]. Furthermore, transient inactivation of NMY-1 and -2 using thermosensitive alleles is reversible, indicating that actin filaments remain intact [79]. While triple *nmy-2*; *spc-1 nmy-*1 loss-of-function embryos have not yet been examined, these observations concur to suggest that myosin-II plays a minor role in actin repair and/or crosslinking.

### 5.3. Anterior–Posterior-Oriented Junction Lengthening

The third process facilitated by muscle activity is the specific lengthening of adherens junctions between seam and non-seam epidermal cells (Figure 5B, step 3), which fail to elongate when muscle-defective embryos arrest at the 2-fold stage [131]. Tracking adherens junction markers using complementary imaging approaches has led to a specific model for how muscle activity promotes anterior–posterior junction extension. Light-sheet microscopy revealed that embryos rotate in their eggshell once muscles begin to contract, and that anterior–posterior junctions alternate between folded and extended states [144]. In parallel, fluorescence recovery after photobleaching (FRAP) analysis showed that muscle activity increases the turnover of HMR-1/E-cadherin at these junctions [144]. Notably, loss of muscle activity increases the immobile fraction of HMR-1 at seam–seam and seam–non-seam junctions by a similar factor (2.5), indicating that muscle activity alone does not explain the preferential extension of anterior–posterior junctions. Instead, a mechanochemical model linking macroscopic deformations to HMR-1 kinetics, together with mathematical modeling of HMR-1 turnover, predicts that the slightly higher HMR-1 concentration observed on anterior–posterior junctions favors their selective lengthening. Consistent with the mechanochemical model predictions, reducing HMR-1 endocytosis through JAC-1/p120catenin knockdown slightly reduces embryo elongation [144]. The mechanochemical model is also in line with other systems showing that elevated E-cadherin concentration and mechanotransduction influence contact expansion by modulating local tension [145,146]. In this context, it would be interesting to determine whether muscle contractions contribute not only to HMR-1 redistribution but also to bursts of HMP-1 unfolding and reinforcement of the actin cytoskeleton at junctions (see Figure 4).

More generally, a comparison between *C. elegans* and *Drosophila* suggests that coupling between tissue-scale deformations and E-cadherin turnover represents a general feature of epithelia undergoing polarized extension through repeated tensional pulses. Strikingly, the repeated pulses occur with similar frequencies in both systems, arising from muscle contractions in *C. elegans* and actomyosin flows in *Drosophila*. In *C. elegans*, embryonic rotations selectively alter the tensional state of anterior–posterior, but not of seam–seam, junctions, promoting HMR-1/E-cadherin enrichment at these interfaces, while circumferential tension stabilizes HMR-1 trans-dimers. In *Drosophila* germband extension and dorsal closure, pulsatile actomyosin contractions and flows drive E-cadherin trafficking [86,87,147].

### 5.4. Planar Polarity and Actin Orientation

The fourth process influenced by muscle activity is the stabilization of actin filaments along the circumferential axis in seam cells (Figure 5B, step 4); these filaments initially lack organization at the onset of elongation (see Figure 3B). As discussed above, the epidermis is planar polarized: actin filaments align circumferentially, and only anterior–posterior junctions extend beyond the 2-fold stage. How might such polarization be established? The PCP pathway is unlikely to play a significant role: although components of the canonical planar cell polarity pathway have been identified in *C. elegans* [26,148], and seem to play a role in VNC morphogenesis, they do not appear to affect embryonic elongation.

A distinct planar-polarized system involves the polarity factors PAR-3/Par3, PAR-6/Pard6, and PKC-3/Prkc, which become restricted to seam–seam junctions after the 2-fold stage [149], coinciding with the onset of muscle activity. Interestingly, *par-3* knockdown embryos display frequent anterior–posterior orientation of actin within seam cells beyond the 2-fold stage. Likewise, in muscle-defective embryos and in embryos lacking CeHD-associated proteins, seam cell actin frequently becomes anterior–posteriorly orientated and PAR-3/PAR-6/PKC-3 retains a bipolar distribution [149]. Together, these observations support a mechanical link between muscle activity and planar polarity in the seam epidermis, which is transmitted through CeHDs and presumably adherens junctions, since *hmp-1* knockdown embryos also show bipolar PAR-3 localization [149]. As discussed in previous sections, actin filaments in dorsoventral epidermal cells may further relay tension from CeHDs to adherens junctions. In contrast to *Drosophila*, where mechanically driven polarity changes also occur between directly adjacent tissues [150], in *C. elegans*, the mechanical signal is relayed across two tissues.

### 5.5. Epidermis-to-Muscle Feedback

Over the past 10–15 years, substantial progress has been made in elucidating the molecular and mechanical mechanisms by which muscle activity drives epidermal morphogenesis. In contrast, much less is known about reciprocal signaling from the epidermis to muscles, despite the requirement that muscles align precisely with the epidermis by the end of elongation to enable coordinated larval crawling. The best-characterized example of such feedback involves SPC-1/α-spectrin, which acts in the epidermis, but the loss of which leads to significant muscle defects [74]. In *spc-1* mutants, body wall muscles are misoriented, fail to undergo normal shape changes, and are circumferentially enlarged. Similar muscle phenotypes are observed in mutants affecting the epidermal actomyosin cytoskeleton, including *sma-1*/β_H_-spectrin, *let-502*/ROCK, and *mlc-4*/MRLC [74]. In addition, mutations in components of CeHDs can locally disrupt muscle organization [138,151]. Together, these observations suggest that epidermis-to-muscle signaling may involve mechanical coupling between basal CeHDs and muscle-dense bodies. Intriguing candidates for mediating this crosstalk could also be ion channels, such as the mechano-sensitive Degenerin/Enac channels UNC-105/Scnn and DEG-1/Scnn, some of which are found at the epidermis–muscle interface (see below). Elucidating the nature of this reciprocal mechanotransduction remains an important challenge for future studies.

## 6. Mechanical Twists on Elongation, Part 3: Coordination of Contractions Among Muscle Cells

Given the prominence of alternating contractions of the left- and right-hand muscle quadrants, which appear to “massage” the embryo (see Figure 5), a natural question arises: what might coordinate such alternating contractions? While neuronal control of coordinated muscle contraction in larvae is well-established [152,153], embryonic muscles are unlikely to rely on the same circuitry since synaptic mutants that paralyze larvae show no elongation defects [154,155]. Although the mechanisms coordinating embryonic muscle activity remain less defined, recent studies have begun to clarify them. First, light-sheet imaging revealed that diagonally positioned muscles tend to contract asynchronously [144]. Second, calcium imaging with GCaMP further showed that contractions initiate dorsally [144,156,157], with the stereotyped activation of five adjacent muscles per quadrant, two of which activate first and most strongly [157] (Figure 5C). A genetic screen identified two innexins (invertebrate homologs of gap-junction connexins) and two Degenerin/Enac channels required for normal contraction patterns and proper embryonic elongation [157]. Because these proteins are known mediators of proprioception and mechanosensation [158,159], they may coordinate muscle contractions in response to stretch. Unexpectedly, one innexin, INX-15 (similar to mammalian Panx1/3), a component of invertebrate gap junctions, acts in the intestine, and the other, INX-18 (similar to mammalian Panx1/3), in muscles (Figure 5C), showing that embryonic elongation relies on mechanical communication across multiple tissues, from intestine to epidermis via muscles [157]. The signals that initiate the very first muscle contraction and those transmitted through intestinal INX-15 remain unknown.

## 7. Twisting the Threads of Elongation Together: Physical Models of Elongation

Physical and mathematical approaches are increasingly informing biological research, including studies of *C. elegans* elongation. Several of the works cited above combine experimental data with biophysical modeling to interpret morphogenetic processes [88,99,109,134,144]. Such hybrid experimental/modeling work has been supplemented by three recent papers that rely exclusively on theory [69,142,160]. All three consider the embryo as a thin-walled cylinder, but differ in their physical emphasis. Using nonlinear elasticity, Ben Amar and colleagues explored how active stresses generated by molecular motors and passive tissue elasticity drive the initial phase of embryonic elongation [69]. They highlight the importance of pre-strain and pre-stress generated as a result of ventral enclosure, as well as evolving embryo geometry when interpreting laser ablation data (see [88]).

Fang and colleagues employed continuum elasticity to examine how cellular anisotropy, plasticity, and force coupling between seam and non-seam cells achieve elongation [142]. By incorporating actin alignment, severing, rebundling kinetics in the epidermis, and muscle activity, they propose that elongation emerges from a mechanochemical feedback acting on a pre-existing anisotropy. Their modeling reproduces key experimental findings for both early and late elongation [88,134].

More recently, Dai and Ben Amar explicitly model the contribution of muscles to elongation using finite and hyperelasticity models [160]. Their key advance is to include the energy storage and dissipative response associated with muscle activity, proposing that embryo rotations (see [144]) generate a torsional bending energy that is converted into finite elongation steps. They also quantify viscous dissipation induced by these rotations.

## 8. Conclusions: Mechanical Twists Are Foundational for *C. elegans* Morphogenesis

**Figure 6:**
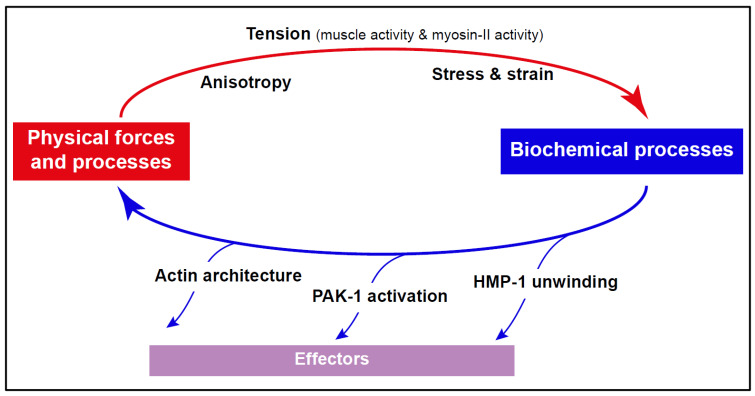
Mechanochemical feedback between molecular states and tissue-level mechanics during morphogenesis. The top part of the diagram (red) illustrates how cell material properties, as well as mechanical forces arising mainly from epidermal myosin-II and muscle activity, modify the stress/strain exerted on tissues, including their orientation (anisotropy). In many instances, the stress/strain exerted at one stage is a precondition for morphogenesis at a later stage, in which case it can be considered a pre-stress/pre-strain. Physical processes, in turn, influence cellular biochemical processes (blue) through mechanosensors; for instance, by modifying the folding/activity/organization of key proteins, which can lead to the activation of entire mechanotransduction pathways (three examples are shown). The corresponding mechanotransduction pathways and their effectors can, in turn, modify the mechanical properties of the cells and, consequently, the state of the physical forces and processes in a constant feedback loop.

*C. elegans* has emerged as a powerful model for understanding how mechanical forces drive changes in cell shape and position during embryonic morphogenesis. Combined molecular genetics, advanced imaging, and biophysical approaches have led to significant progress in this field (see Box 2). A key insight emerging from the work reviewed here is the existence of feedback mechanisms between biochemical processes at the level of individual proteins and emergent mechanical forces at the level of tissues or the entire embryo. In particular, mechanical forces help coordinate the morphogenesis of multiple tissues. One of the best-understood examples of such feedback occurs during embryonic elongation. In this process, changes in the state or activity of proteins that respond to mechanical load (e.g., HMP-1, PAK-1, or actin architecture) propagate through downstream effectors to alter the bulk mechanical properties of cells and the anisotropic forces generated by whole tissues. These forces, in turn, feed back onto the force-dependent states of mechanosensitive proteins (Figure 6).

Identifying mechanosensitive feedback during other morphogenetic events is a challenge for the future. Several additional key questions remain unresolved. First, how is circumferential planar polarity established in the epidermis, and what are the relative roles of early tension, acting to pre-stress the epidermis, versus PCP regulators? Second, related to the first question, how are actin filaments (CFBs) in dorsoventral bundles organized, and what makes epidermal cells more compliant in the direction of elongation? Third, how might the diversity of actin isoforms and non-muscle myosin heavy-chain isoforms modulate the mechanical properties of the embryo? Fourth, how, at a mechanistic level, does muscle activity influence adherens junction remodeling, and, conversely, how does the epidermis regulate muscle biogenesis? Fifth, what is the potential contribution of the eggshell to elongation (see [160])? Finally, since morphogenetic programs are tightly integrated with concomitant changes in gene expression, how do gene regulatory networks (GRNs) regulate morphogenesis by modulating transcriptional targets? Moreover, given the high level of functional redundancy observed in embryos (see sections above), recent genome in situ expression screens and genome profiling studies may help identify new genes that modulate embryonic processes [161,162,163] (see also Box 2). Stage-specific profiling of larval tissues, for instance, is identifying important genes [164,165], although such studies would be significantly more difficult to carry out at the embryonic elongation stage due to the lack of good time- and tissue-specific markers.

Answering these questions will require new approaches. As discussed in this review, genetic studies have revealed a high degree of redundancy in the control of most morphogenetic processes, which may be a general feature across species. The use of optogenetics to precisely assess the spatiotemporal effects of individual regulators should help tease apart their respective contributions. Although imaging methods are becoming increasingly powerful, localized biophysical perturbation techniques remain difficult to implement in vivo, partly due to the eggshell and the small size of *C. elegans* embryos. Improved approaches for monitoring intracellular protein activity, such as FRET-based biosensors and kinase activity reporters, should help define when specific proteins are recruited or become active. Finally, there is an urgent need to bridge theoretical models and experimental approaches, so that transition points in phase diagrams used by theorists are well-grounded in real-world parameter space [166,167]. Given the rapid pace of technological innovation in the *C. elegans* community, there is every reason to believe that these and other technological barriers can be overcome.

Box 1 **List of abbreviations and short lexicon of physical terms.**

List of abbreviations. **CeAJ—*C. elegans* apical junction:** adhesive complex containing the cadherin/catenin complex proteins, the **CCC**, and the DLG-1/AJM-1 protein complex, the **DAC**. **CeHDs—*C. elegans* hemidesmosome-like junctions**: hemidesmosome-like junctions ensuring epidermis attachment to the shared extracellular matrix between muscles and the epidermis, and to the overlying apical extracellular matrix; they are also referred to as fibrous organelles due to their appearance in electron micrographs. **CFB—circumferential filament bundle**: parallel bundle of F-actin filaments in the dorsal and ventral embryonic epidermis of *C. elegans*.

Lexicon of some physical terms. **Catch-bond**: an interaction between proteins that becomes stronger or longer under moderate amounts of force. **Isotropic and anisotropic**: refer to material properties of an object that are the same in all directions or different in different directions, respectively. **Kelvin–Voigt material**: a material corresponding to a spring in parallel to a dashpot, which are together attached on one end and submitted to tension on the other; it is the simplest model of a viscoelastic material in which the elastic spring force is dampened by the dashpot. **Mechanosensor**: a protein embedded in a large cell structure that detects changes in forces applied to the structure. **Mechanotransduction**: A signal transduction process downstream of a mechanosensor in response to changes in forces. **Strain**: relative change in size of an object. **Stress**: ratio of the force to the area of contact.

Box 2 **New approaches for studying embryonic morphogenesis.**

Over the past 10–12 years, numerous new approaches have been introduced to study morphogenesis and its molecular determinants. When combined with the genetic analysis enabled by C. elegans, these approaches have proven powerful. Below, we survey some of them.

Light-Sheet Fluorescence Microscopy (LSFM): LSFM relies on two separate and orthogonally arranged lenses for excitation and collection of emitted fluorescence. Compared with traditional confocal microscopy, LSFM also provides optical sectioning, while reducing out-of-focus illumination and sample photo-damage, enabling long-term imaging [168]. Variants of LSFM, such as dual-view plane illumination microscopy or lattice light-sheet microscopy, further improve the spatiotemporal resolution [169,170]. LSFM generates very large datasets, making image analysis a challenge (see below).

Microscopy Image Analysis: Several investigators have proposed protocols to analyze and process LFSM and large datasets from other microscopy modes. They generally require (1) reducing background noise using imaging filters, potentially deconvolving images to improve resolution, then (2) segmenting and tracking images (for an in-depth review, see [171]). Several open-source methods and atlases are available to help analyze images from moving embryos [172,173,174]. More recent approaches rely on deep learning [55,175].

Force Measurement: Three methods have been used to assess morphogenetic forces in recent years. First, tracking the recoil velocity of cytoskeletal filaments or junctions after their severing using a laser provides information regarding the tension they bear. This can be achieved using either a UV laser [176] or the infrared laser of two-photon microscopes [88] (for a review on the respective merits of each laser source, see [177]).

A second in vivo method relies on the expression of genetically encoded FRET tension sensors. In this method, a protein that undergoes a conformational change upon stretching is engineered to contain two distinct fluorophores separated by a flexible linker that are positioned on either side of the force-sensitive domain [178]. This strategy has been used in *C. elegans* larvae to assess the tension borne by β-spectrin in axons [179], and by α-catenin/HMP-1 in the embryonic epidermis [97]. While appealing, and capable in principle of measuring pN forces, this approach is labor-intensive as it requires the generation of multiple control constructs and careful in vitro calibration of the FRET sensor probe when inserted into a new recipient protein.

A third approach relies on in vitro single-molecule force spectroscopy measurements using magnetic tweezers to investigate the force required to unfold a particular domain [137]. This method has been applied to quantify the mechanical stability of full-length α-catenin/HMP-1 and its force-bearing modulation domains (M1–M3), as well as the β-catenin/HMP-2–HMP-1 interface [99]. This approach is technically demanding and typically requires collaboration with experienced biophysicists.

Tools to Manipulate Protein Activity: In recent years, numerous tools have been developed to selectively eliminate a specific protein, selectively activate protein activity, or promote protein interactions in a given cell. While these techniques were initially developed to manipulate synaptic activity, they are increasingly being adapted to other cell types.

Here, we briefly mention a few of these approaches. Protein degradation has been achieved by repurposing endogenous protein degradation signals used in the early embryo or by adapting the auxin-inducible degradation system of plants [180,181]. Selective protein–protein interaction has been achieved using the ePDZ–LOV cassette in the early *C. elegans* embryo [182,183,184]. An alternative method to control protein–protein interaction through light relies on the CRY2-CIB1 cassette [185]. Seminal papers on the use of these cassettes can be found here [186,187,188,189,190].

Modeling Morphogenetic Processes: Because embryonic morphogenesis relies heavily on mechanical forces, it is important to understand it from a physical perspective. The appropriate theoretical framework depends in part on the specific question being addressed. Nonlinear and finite elastic theories, hyperelastic models, as well as mechanochemical frameworks have all been used to account for cellular anisotropy, plastic deformation, and embryonic morphogenesis driven by non-muscle myosin and muscle-generated forces [69,142,144,160]. Additionally, mathematical analysis has been used to analyze laser dissection or fluorescence recovery after photobleaching (FRAP) data [88,144]. Each of these physical theories puts the emphasis on different concepts and entities, specifically: large or small finite deformations treated with deformation gradients with relationship to geometry (nonlinear and finite elasticity); stored energy potential and its influence on stress (hyperelastic theory); free energy arising from the coupling between mechanical deformation and chemical processes (mechanochemical theory) (for textbooks on these models, see [191,192,193]).

Large-Scale Screens: Progress in imaging and image analysis has facilitated large-scale genome-wide screening to identify novel genes involved in morphogenesis [161]. Progress in single-cell sequencing and genome analysis has likewise allowed the connection of gene expression with cell lineages over time [163].

## Figures and Tables

**Table 1:** Homologs of *C. elegans* proteins mentioned in the figures.

Figure	C. elegans *	Mouse Homolog **	Presumed or Known Function ***
1	UNC-5	Unc5	Netrin receptor
	TIAM-1	Tiam1	Rac guanine-nucleotide exchange factor
	UNC-73	Trio	Rac guanine-nucleotide exchange factor
	CED-10	Rac1	Rho-family small GTPase
	MIG-2	RhoG	Rho-family small GTPase
	VAB-1	EphB2/EphA4	Ephrin family tyrosine kinase receptor
	CDC-42	Cdc42	Small GTPase
	UNC-40	Dcc	Netrin receptor
	SAX-3	Robo	Ig-repeat family transmembrane receptor involved in axon guidance
	ECT-2	Arhgef31	Rho guanine-nucleotide exchange factor
	HUM-7	Myosin-IXa	Unconventional myosin with RhoGAP domain
	CED-5	Dock180	Bipartite Rac guanine-nucleotide exchange factor with CED-12
	CED-12	Elmo	Bipartite Rac guanine-nucleotide exchange factor with CED-5
	RGA-8	Sh3bp1/Rich1/Arhgef17	Rac/Cdc42 GTPase-activating protein
	SPV-1/RGA-7	Arhgap29	F-BAR Cdc42 GTPase-activating protein
	TOCA-1	Fnbp1/Trip10	F-BAR domain Cdc42 effector
	RHO-1	Rho1	Rho-family small GTPase
	LET-502	Rho-binding kinase	Protein kinase
	NMY-2	Myh10/Myh11	Non-muscle myosin
	WSP-1	Wasp	Actin nucleation-promoting factor
	WVE-1	Wave1	Wiskott–Aldrich syndrome branched actin nucleator
2	MIG-1	Frizzled-4	7-pass transmembrane family Wnt receptor
	VANG-1	Vang1	4-pass transmembrane protein regulating Wnt signaling
	HMP-1	α-catenin	Core adherens junction component
	AFD-1	Afadin	Actin-binding cell adhesion protein
	SRGP-1	Arhgap4	F-BAR Rho GTPase-activating protein
	PAR-6	Pard6a/g	Cell polarity adaptor factor
	DYF-7	No homolog	Zona Pellucida transmembrane protein
	HMR-1	E-cadherin	Core adherens junction component
	DLG-1	Dlg2	MAGUK-family cell adhesion protein
	SAX-7	L1cam	Cell adhesion transmembrane protein
3	NMY-1	Myh9/Myh10	Non-muscle myosin heavy chain
4	HMP-2	ß-catenin	Core adherens junction component
	MAGI-1	Magi2/3	MAGUK-family scaffolding protein
	AJM-1	Ajm1	Apical junction component
	TES-1	Testin	LIM-domain actin-binding protein
	UNC-94	Tropomodulin	Pointed-end actin-capping protein
	ZYX-1	Zyxin	LIM-domain actin-binding protein
	DEB-1	Vinculin	Actin- and α-catenin-binding protein
	JAC-1	p120-catenin	Juxtamembrane domain-associated catenin
	SRGP-1	Arhgap4	F-BAR Rho GTPase-activating protein
	PICC-1	Ccdc85a/b/c	PAC-1 interacting and coiled-coil protein
	PAC-1	Arhgap1/23	Cdc-42-specific RhoGAP
	ZOO-1	Tight junction protein Tjp2	MAGUK cell–cell adhesion protein
	VAB-9	Bcmp1	4-pass transmembrane protein of the Claudin family
5	GIT-1	Git1	ARF GTPase-activating protein
	PIX-1	Arhgef6/7	Rac GTPase-activating protein
	PAK-1	Pak1	p21-activated Ser/Thr protein kinase
	VAB-19	Kank family	Ankyrin-domain adhesion protein
	EPS-8	Eps8	SH3-domain actin-binding protein
	VAB-10	Plectin/Macf	Cytolinker protein
	MUP-4	No homolog	Transmembrane adhesion protein with extracellular EGF/VWA/SEA domains
	UNC-52	Perlecan	Basement membrane protein
	LET-805	No homolog	Transmembrane adhesion protein with multiple extracellular FN domains bearing similarity to Tenascin
	INX-15	Innexin	4-pass transmembrane protein related to connexin
	INX-18	Innexin	4-pass transmembrane protein related to connexin
	IFA-3	Related to Lamins	Cytoplasmic intermediate filament dimerizing with IFA-3
	IFB-1	Related to Lamins	Cytoplasmic intermediate filament dimerizing with IFB-1
	SPC-1	α-spectrin	Cytoskeletal scaffolding protein
	FHOD-1	Fhod1	Formin
	VILN-1	Villin	Actin-severing protein
	GSNL-1	Gelsolin	Actin-severing protein
	PAR-3	Par3	Cell polarity adaptor protein
	PKC-3	Pkc3	Cell protein Ser/Thr protein kinase

* *C. elegans* proteins as indicated in figures; details about a given protein are provided only once, even if it appears in more than one figure. ** The names of the closest mouse protein homologs are indicated. *** Function is inferred either from the *C. elegans* or the vertebrate literature (see text).

**Table 2:** Sequence of the main events discussed in the text and figures.

Event	Stage *	Figures	Text section
Cell migrations	180 cells/4 h	2A	Sequential rosettes
Dorsal intercalation	240 cells/5 h	1A	Dorsal intercalation
Ventral cleft closure	330 cells/5.5 h	2B	Sequential rosettes
Ventral enclosure	300–360 cells/5.5 h	1B	Ventral enclosure
Ventral nerve cord formation	360 cells/6 h	1C	Nerve cord assembly through convergent extension
Head morphogenesis	430 cells/6,5 h	1D	Anterior morphogenesis
Elongation	360–550 cells/6.5–9.5 h	3 and 4	Part 1 and part 2 of Mechanical twists on elongation
Influence of muscles on the epidermis	550 cells/8–9.5 h	5	Part 2 of Mechanical twists on elongation

* Stages indicate total cell number and time after fertilization (in hours) at which the process begins at 20 °C; beginning and end stages are provided for particularly long processes.

## Data Availability

No new data were created or analyzed in this study.
